# Targeted next-generation sequencing panels in the diagnosis of Charcot-Marie-Tooth disease

**DOI:** 10.1212/WNL.0000000000008672

**Published:** 2020-01-07

**Authors:** Andrea Cortese, Janel E. Wilcox, James M. Polke, Roy Poh, Mariola Skorupinska, Alexander M. Rossor, Matilde Laura, Pedro J. Tomaselli, Henry Houlden, Michael E. Shy, Mary M. Reilly

**Affiliations:** From the MRC Centre for Neuromuscular Diseases (A.C., J.M.P., R.P., M.S., A.M.R., M.L., P.J.T., H.H., M.M.R.), Department of Neuromuscular Diseases, National Hospital for Neurology and Neurosurgery, UCL Queen Square Institute of Neurology, London, UK; Department of Brain and Behavioral Sciences (A.C.), University of Pavia, Italy; and the Department of Neurology (J.E.W., M.E.S.), University of Iowa Carver College of Medicine, Iowa City.

## Abstract

**Objective:**

To investigate the effectiveness of targeted next-generation sequencing (NGS) panels in achieving a molecular diagnosis in Charcot-Marie-Tooth disease (CMT) and related disorders in a clinical setting.

**Methods:**

We prospectively enrolled 220 patients from 2 tertiary referral centers, one in London, United Kingdom (n = 120), and one in Iowa (n = 100), in whom a targeted CMT NGS panel had been requested as a diagnostic test. *PMP22* duplication/deletion was previously excluded in demyelinating cases. We reviewed the genetic and clinical data upon completion of the diagnostic process.

**Results:**

After targeted NGS sequencing, a definite molecular diagnosis, defined as a pathogenic or likely pathogenic variant, was reached in 30% of cases (n = 67). The diagnostic rate was similar in London (32%) and Iowa (29%). Variants of unknown significance were found in an additional 33% of cases. Mutations in *GJB1*, *MFN2*, and *MPZ* accounted for 39% of cases that received genetic confirmation, while the remainder of positive cases had mutations in diverse genes, including *SH3TC2*, *GDAP1*, *IGHMBP2*, *LRSAM1*, *FDG4*, and *GARS*, and another 12 less common genes. Copy number changes in *PMP22*, *MPZ*, *MFN2*, *SH3TC2*, and *FDG4* were also accurately detected. A definite genetic diagnosis was more likely in cases with an early onset, a positive family history of neuropathy or consanguinity, and a demyelinating neuropathy.

**Conclusions:**

NGS panels are effective tools in the diagnosis of CMT, leading to genetic confirmation in one-third of cases negative for *PMP22* duplication/deletion, thus highlighting how rarer and previously undiagnosed subtypes represent a relevant part of the genetic landscape of CMT.

Charcot-Marie-Tooth disease (CMT) and related disorders distal hereditary motor neuropathy (dHMN) and hereditary sensory neuropathy (HSN) represent the most common heritable neurologic conditions and to simplify discussion all 3 diseases are referred to as CMT in this article.^1^ To date, over 90 genes have been associated with CMT and the number is increasing.^2^

Despite the genetic heterogeneity underlying CMT, to date up to 90% of all genetically confirmed cases across different cohorts are reportedly due to mutations in only 4 genes—*PMP22* duplication/deletion and mutations in *PMP22*, *GJB1*, *MFN2*, and *MPZ*—while up to 40% of patients remain genetically undiagnosed.^3–5^ This is not surprising if one considers that, until recently, molecular diagnosis of CMT has relied on multiplex ligation-dependent probe amplification (MLPA) for chr17p12 and conventional Sanger sequencing of these major causative genes.

In recent years, genetic diagnosis of inherited diseases has evolved rapidly with the advent of next-generation sequencing (NGS). NGS technology allows multiple parallel sequencing of the whole human genome (whole genome sequencing), its protein coding sequences (whole exome sequencing [WES]), or specific genes of interest (targeted multigene panels).

Application of WES to molecularly undefined families with CMT has enabled the recent exponential growth in discovery of genes associated with CMT. WES has also proved effective in the screening of patients with CMT for known genes, achieving a molecular diagnosis in 9%–45% of cases, depending on the characteristics of the cohort and the criteria used for classifying causative mutations.^6–9^ This notwithstanding, implementation of WES in the diagnostic practice is hampered by its suboptimal gene coverage, as well as the large volume of data generated.

In recent years, customized targeted NGS panels of disease-relevant genes have been the preferred method for employing NGS in clinical practice and offer a high degree of coverage of the selected genes.

Several groups have published their use of custom NGS panels in the diagnosis of CMT in a research setting and validated its efficacy in detecting point mutations, small insertions, and deletions as well as larger rearrangements, including the common chr17p12 duplication causing CMT1A.^10–16^ Nevertheless, there are limited data on the effect of targeted NGS panels on the genetic diagnosis of CMT in everyday clinical practice.^17–20^ This study describes the effect of targeted NGS panels on the molecular diagnosis of CMT and related disorders in routine clinical practice in 2 specialized clinics in different health systems in the United Kingdom (London) and United States (Iowa).

## Methods

### NGS panels

In London, NGS was performed by the National Hospital for Neurology and Neurosurgery's (NHNN) United Kingdom Accreditation Service–accredited genetic diagnostic laboratory where 50 genes associated with CMT and related conditions were sequenced (uclh.nhs.uk/OurServices/ServiceA-Z/Neuro/NEURG/NGLAB/Pages/UCLHNeurogeneticspanels.aspx). Subgroups of the panel can also be ordered for phenotype-driven targeted sequencing (e.g., HSN) varying from 11 to 50 genes. In the NHNN laboratory, enrichment was performed with an Illumina (San Diego, CA) custom Nextera Rapid Capture panel prior to NGS on an Illumina MiSeq, Hiseq 2500, or NextSeq 500. All coding exons of the RefSeq transcripts of the genes and 15 base pairs of the flanking introns were targeted, except for *GJB1*, for which the target region is extended 860 bases upstream of the ATG start codon to include the nerve-specific promoter region, and *NTRK1*, for which the targeting is extended to include the known splicing mutation c.851-33T>A. Variants that are pathogenic, likely to be pathogenic, and of uncertain clinical significance were confirmed by bidirectional Sanger sequencing. Over 99% of the coding exons of all genes in the panel were sequenced to a read depth of 30× or greater in almost all cases.

In Iowa, NGS study was outsourced to accredited commercial companies, which returned detailed information on 51 ± 23 (18–135) sequenced and analyzed genes.

A full list of genes sequenced and analyzed in London and Iowa is provided in table e-1 (doi.org/10.5061/dryad.kp8pb51). Mutations were classified according to the 2015 American College of Medical Genetics Standards and Guidelines for the interpretation of sequence variants^21^ and cases with pathogenic or likely pathogenic mutations were considered as genetically confirmed. All cases were discussed after NGS testing in a multidisciplinary setting including CMT specialist neurologists, geneticists, genetic counselors, neurophysiologists, and neuropathologists, where appropriate.

### Data collection and statistical analyses

Patients with CMT attending specialized inherited neuropathy clinics in both centers were enrolled from January 2015 to December 2017. Patients were diagnosed with CMT based on the presence of a slowly progressive neuropathy with or without family history and after exclusion of other common causes of acquired neuropathy. After review of clinical charts, the following information was recorded for all patients: age at NGS testing (enrollment), age at onset, sex, family history of neuropathy or consanguinity, symptoms at onset, additional phenotype, and motor conduction velocity of nondominant median or ulnar nerve during first nerve conduction study available. CMT subtype was classified as CMT if both motor and sensory nerves were similarly affected, and dHMN or HSN if the neuropathy showed exclusive or predominant involvement of motor or sensory nerves, respectively. CMT cases were further subdivided into demyelinating CMT if conduction velocity of the nondominant median or ulnar nerve was ≤38 m/s and axonal or intermediate CMT if >38 m/s. Disease severity was scored using the previously validated Charcot-Marie-Tooth neuropathy score (CMTNS, v2) or CMT examination score (CMTES, v2)^22^ and cases were divided into mild (CMTNS 0 to 10 or CMTES 0 to 7), moderate (CMTNS 11 to 20 or CMTES 8 to 16), and severe (CMTNS 21 to 36 or CMTES 17 to 28). In cases for which CMTNS or CMTES had not been collected, disease was considered mild if walking was possible without aid, moderate if walking was possible with foot orthosis or ankle dorsiflexion was <3 Medical Research Council grade, and severe if patients needed a walking aid, such as a stick or a wheelchair. Continuous data are shown as mean ± SD. Differences between groups were determined with 2-tailed *t* test for quantitative variables, with χ^2^ test for categorical variables, as appropriate. A multivariate logistic regression was performed to assess the association of relevant clinical variables with a positive result of targeted NGS testing. Pearson correlation coefficient was calculated to test association of presence or number of variants of unknown significance (VUS) and disease severity. All analyses were performed using STATA statistical software, version 14.

### Standard protocol approvals, registrations, and patient consents

The study was approved by local institutional ethical committees. Written informed consent was obtained from all patients (or guardians of patients) participating in the study.

### Data availability

Anonymized data from this study will be shared by request from any qualified investigator.

## Results

### Patient cohorts

A total of 220 consecutive patients with CMT were enrolled from January 2015 to December 2017 in London (n = 120) or Iowa (n = 100). Relevant demographic and clinical features are summarized in table 1. Sixty-one percent were male and mean age at enrollment was 49 ± 17 years. The most frequent CMT subtype in enrolled cases was axonal or intermediate CMT (n = 143, 65%), followed by demyelinating CMT (n = 41, 19%), dHMN (n = 21, 9%), and HSN (n = 15, 7%). The proportion of patients with dHMN and HSN was higher in London than Iowa, where the majority of cases had axonal or intermediate CMT; however, there was no significant difference in the proportion of patients with demyelinating CMT. *PMP22* duplication/deletion was excluded in all patients with typical demyelinating CMT prior to NGS testing. Twenty patients had an independent risk factor for neuropathy including diabetes (n = 9), paraprotein (n = 4), previous chemotherapy (n = 2), rheumatoid arthritis (n = 2), Sjögren syndrome (n = 1), high alcohol consumption (n = 1), or renal transplant (n = 1). In 16 of the 220 cases, a diagnosis of CMT was considered the most likely diagnosis justifying genetic testing but was not definite. This reflects real-life clinical practice. In Iowa, there was a higher percentage of familial cases, but the number of cases with onset of the neuropathy before 20 years of age was lower compared to the London cohort. Patients in London more frequently had undergone previous genetic testing by Sanger sequencing of candidate genes compared to patients in Iowa, likely due to insurance coverage restrictions in the United States.

**Table 1:**
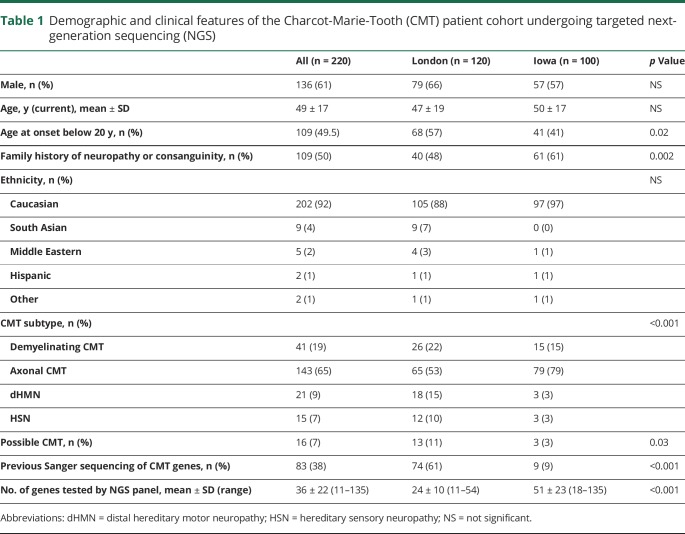
Demographic and clinical features of the Charcot-Marie-Tooth (CMT) patient cohort undergoing targeted next-generation sequencing (NGS)

### Genetic diagnosis

**Figure 1:**
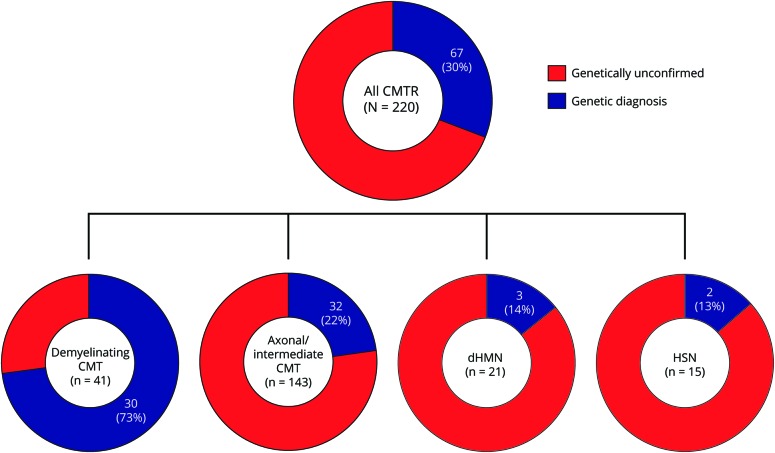
Distribution of patients receiving genetic diagnosis according to Charcot-Marie-Tooth disease (CMT) subtype CMTR = Charcot-Marie-Tooth disease and related disorders; dHMN = distal hereditary motor neuropathy; HSN = hereditary sensory neuropathy.

After targeted NGS sequencing, a genetic diagnosis, defined as a pathogenic or likely pathogenic variant, was reached in 30% of cases (n = 67) (tables 2 and e-2 [doi.org/10.5061/dryad.kp8pb51]). The diagnostic rate was similar in London (32%) and Iowa (29%). The proportion of cases with a genetic diagnosis was higher for demyelinating CMT (n = 30/41, 73%) compared to axonal or intermediate CMT (n = 32/143, 22%), dHMN (n = 3/21, 14%), or HSN (n = 2/15, 13%) (figure 1). Overall, variants in *GJB1* (n = 12), *SH3TC2* (n = 8), *MFN2* (n = 8), and *MPZ* (n = 6) accounted for half of the genetically confirmed patients, followed by *GDAP1* (n = 4), *IGHMBP2* (n = 4), *LRSAM1*, *FDG4* and *GARS* (n = 3 per gene), *AARS*, *LITAF*, and *PMP22* (n = 2 per gene). Nine patients had mutations in an additional 9 different genes.

**Table 2:**
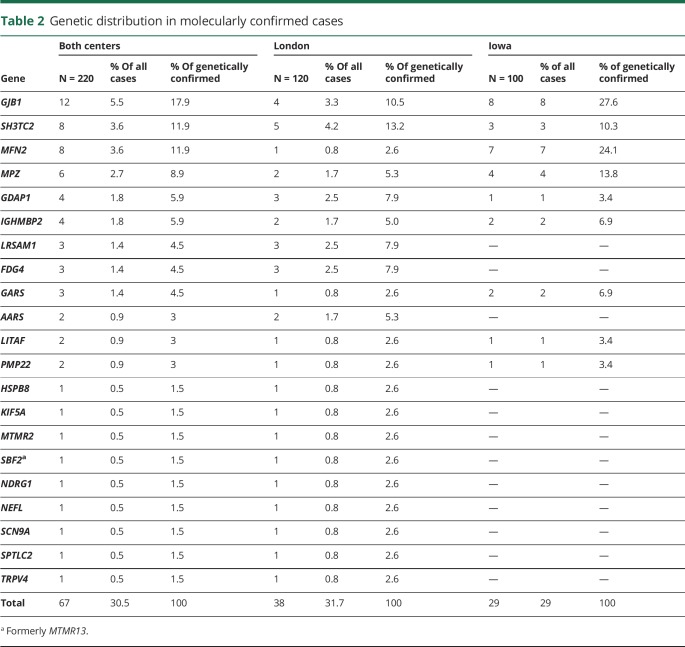
Genetic distribution in molecularly confirmed cases

In Iowa, mutations in *GJB1*, *MPZ*, and *MFN2* accounted for 66% of genetically confirmed cases (n = 19), followed by *SH3TC2* (3 cases) and 5 less common genes.

In London, only 18% of solved cases had a mutation in *GJB1*, *MPZ*, or *MFN2* (n = 7). Five patients had mutations in *SH3TC2*. Mutations in *LRSAM1*, *FDG4*, *GDAP1*, *AARS*, and *IGHMBP2* collectively accounted for a third of genetically confirmed patients.

Copy number variants were identified in 7 patients (3%) and in 5 of them were considered pathogenic or likely pathogenic including whole gene deletion of *PMP22*, whole gene duplication *of MPZ*, exonic deletions of *MFN2* (exons 7 and 8), *SH3TC2* (exon 7), and a 90–base pair deletion in exon 5 of *FDG4.*

Overall, 30 pathogenic or likely pathogenic mutations were novel.

### Predictors of positive targeted NGS testing

Patients who received a genetic diagnosis after targeted NGS sequencing were more likely to have an earlier age at onset, a positive family history, or a demyelinating neuropathy. These variables were confirmed to be independent predictive factors of positive NGS testing in a multivariate logistic regression model (table 3). In only 1 out of 16 patients with a low pretest probability of having CMT did NGS testing yield a positive result. Sex, number of genes present on NGS panels, and disease severity were not associated with achievement of a genetic diagnosis.

**Table 3:**
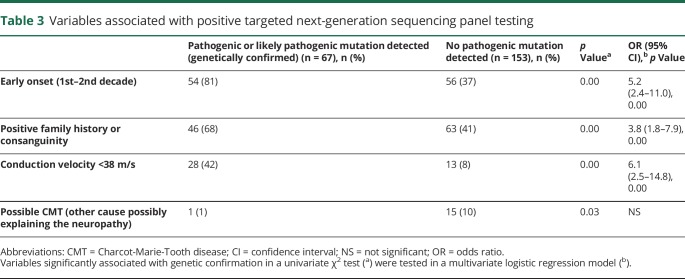
Variables associated with positive targeted next-generation sequencing panel testing

### Ancillary testing

**Figure 2:**
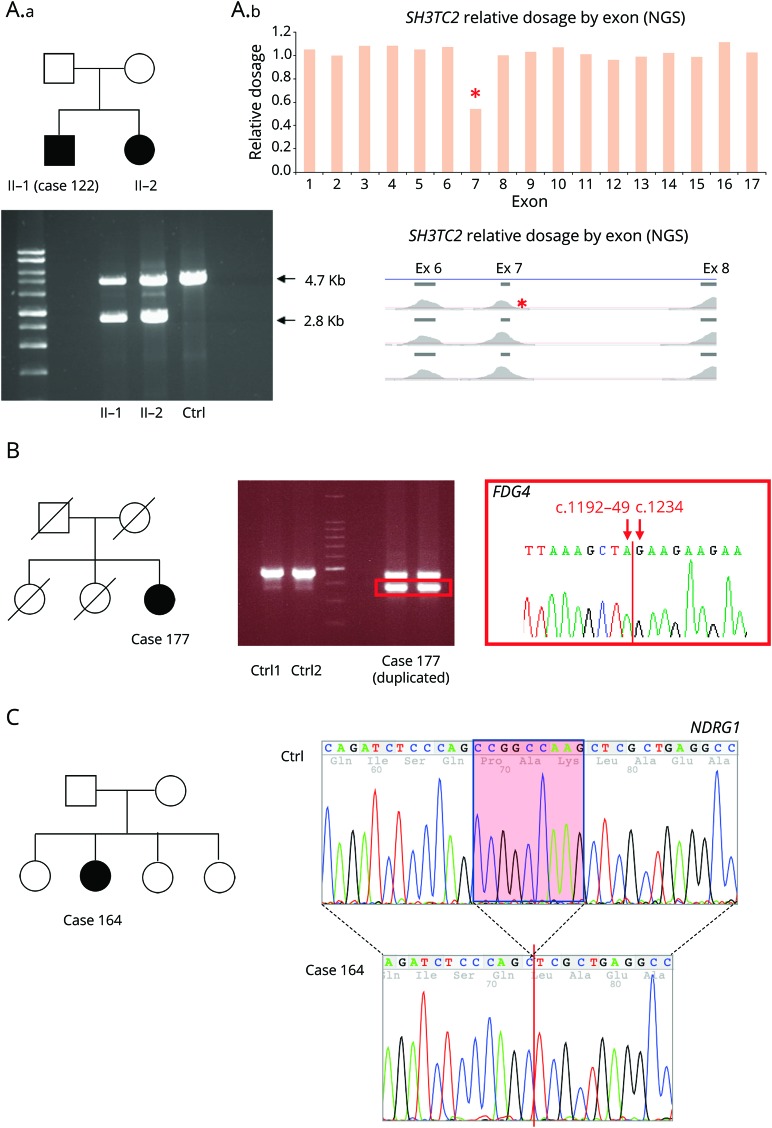
Representative examples of ancillary testing to next-generation sequencing (NGS) performed in selected cases (A) Case 122 presented with early onset of demyelinating neuropathy associated with scoliosis and cranial nerve involvement. He had a sister with a similar condition. NGS for genes associated with Charcot-Marie-Tooth disease type 1 (CMT1) and intermediate was performed and identified a single c.386-2A>C mutation in *SH3TC2*. Relative read-depth analysis of NGS was performed (A.b) looking for copy number variant in *SH3TC2* and identified a deletion of exon 7 (indicated by a red * on the read depth plots), which was confirmed by long PCR in both siblings (A.a), in compound heterozygous state with the c.386-2A>C. (B) Patient 139 was diagnosed in the first decade of life with CMT1. A targeted NGS panel was performed at age 72, which identified 2 variants in FDG4 1304_1305delinsAA p.(Arg435Gln) and FDG4:c.1192-48_1233del. Long-range PCR was performed followed by Sanger sequencing of the gel band-extracted PCR product (red square box) identifying the breakpoints of 90–base pair FGD4 deletion. (C) Patient 164 presented with early-onset CMT1. NGS targeted panel for *CMT1* genes was performed at age 35 and identified a homozygous 892-1 G>T variant in *NDRG1*, bearing potential to disrupt splicing of the flanking exons. RNA was extracted from peripheral blood and retrotranscribed into cDNA showing that the splicing mutation leads to a 9–base pair deletion of *NDRG1* transcript (c.892_900delCCGGCCAAG) resulting in an in-frame deletion of 3 amino acids (red box). As opposed to typical CMT4D cases due to stop mutations in *NDRG1*, patient 164 presented a relatively mild neuropathy without clinical evidence of hearing loss, suggesting that the splicing mutation leading to in-frame deletion of 3 amino acidic residues may not abolish *NDRG1* function.

In order to confirm or reject a variant found by NGS as causative of the neuropathy, additional investigations were performed in 57 cases (26%), including 25 cases (21%) in London and 32 cases in Iowa (32%). Segregation analysis was the most common ancillary investigation performed (51 cases). Long-range PCR followed by Sanger sequencing or MLPA were performed in 5 cases to confirm large rearrangements, including whole gene deletion of *PMP22* and whole gene duplication of *MPZ*, exonic deletion of *MFN2* (exons 7 and 8), *SH3TC2* (exon 7) (figure 2A), and a 90–base pair deletion in exon 5 of *FDG4* (figure 2B). RNA studies were performed in order to determine the effect of a novel homozygous 892-1 G>T variant in *NDRG1* on splicing. cDNA from RNA for peripheral blood showed that the splicing mutation leads to a 9–base pair deletion (c.892_900delCCGGCCAAG) resulting in an in-frame deletion of 3 amino acids (figure 2C). In vitro studies were performed to gather additional functional evidence of pathogenicity of a noncoding mutation in the 3′UTR of *GJB1*^23^ and to test the loss-of-function effect of a novel variant in *AARS* in a yeast aminoacylation complementation assay. Plasma concentrations of 1-deoxy-sphinganine and 1-deoxymethyl-sphinganine are currently being measured in 3 cases with variants in *SPTLC1* and *SPTLC2*.

### Variants of unknown significance

Ninety-eight VUS were found in 73 patients, including 52 cases for which no other pathogenic or likely pathogenic variant could be identified (table 4). Heterozygous variants in *SH3TC2*, *NTRK1*, *PRX*, *NGF*, and *PLEKHG5* and other genes associated with autosomal recessive CMT accounted for half of cases. VUS were also found frequently in *AARS*, *DYNC1H1*, and *SPTLC1* and their interpretation was often challenging. Eighteen cases had more than one VUS. A detailed list is provided in table e-3 (doi.org/10.5061/dryad.kp8pb51). Multiple reasons prevented the interpretation of a VUS as causative of the neuropathy, including the presence of the variant in public healthy control databases (20 cases, 27%), novel variants (17 cases, 23%), weak in silico predicted scores of pathogenicity (10 cases, 15%), poor conservation of the mutated amino acid across species (7 cases, 9%), and a lack of functional evidence of pathogenicity (8 cases, 11%). Evaluation of the clinical phenotype was used to exclude a VUS as pathogenic in 27 patients (36%).

**Table 4:**
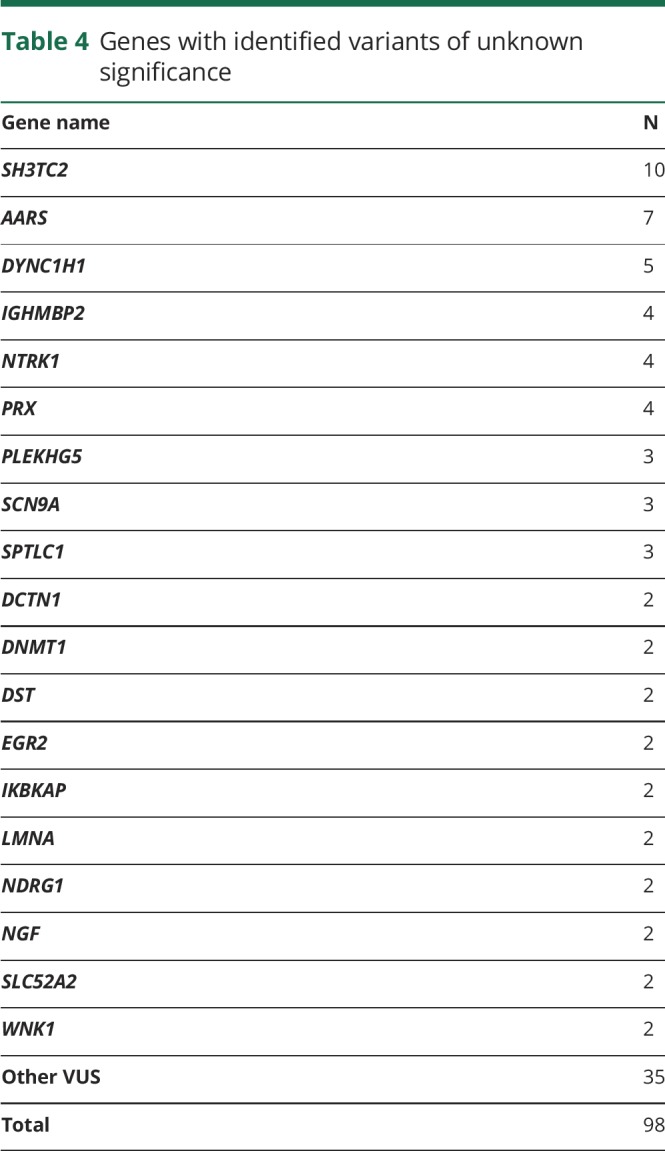
Genes with identified variants of unknown significance

There was no significant difference in the presence of VUS identified in patients with (31%, n = 21) or without (34%, n = 52) a definite molecular diagnosis (Pearson χ^2^ = 0.15, *p* value = 0.7), number of VUS in patients with (1 VUS in 27%, n = 18; 2 VUS in 3%, n = 2; 3 or more VUS in 3%, n = 2) or without (1 VUS in 25%, n = 39; 2 VUS in 7%, n = 10; 3 or more VUS in 3%, n = 4) a definite molecular diagnosis (Poisson χ^2^ = −0.16, *p*-value = 0.76), nor was there a correlation between the presence (Pearson correlation coefficient = −0.06, *p* value = 0.6) or number (Pearson correlation coefficient = −0.16, *p* value = 0.2) of additional VUS and disease severity in genetically confirmed patients.

### Clinical features of the most common genetic CMT subgroups

Mutations in *GJB1*, including 3 mutations in noncoding regions of the gene, 2 in the promoter, and 1 in the 3′UTR, were found in 6 male and 6 female participants, thus representing the most common genetic subgroup identified (17% of solved cases). The clinical features were similar to those described previously.^24,25^ Male participants had onset in the first or second decade, disease severity was more frequently moderate, and conduction velocity in the intermediate to demyelinating range. Female participants had onset in the second or third decade. In female participants, conduction velocities were normal in 2 cases, reduced in the intermediate range in 3, and slow in the *CMT1* range in 1. Disease severity was variable.

With 8 cases diagnosed, recessive mutations in *SH3TC2* accounted for 12% of solved CMT cases and 27% of the demyelinating CMT subtype. Patients were all of Caucasian origin, symptom onset was usually reported in the first decade, and motor milestones were frequently delayed. Scoliosis was observed in 6 cases and cranial nerve involvement was reported in 3. However, progression of the neuropathy was generally slow, and its severity moderate. Conduction velocities were reduced, ranging from 20 to 33 m/s. An illustrative case of the utility of unbiased parallel sequencing followed by segregation analysis vs candidate-gene direct sequencing in interpreting single variants is highlighted by the case of 2 affected brothers with CMT1 who were previously given a diagnosis of CMT1C due to a novel c.115C>T p.(Pro39Ser) mutation in *LITAF* identified by Sanger sequencing. The affected patients had early scoliosis and deafness, which is unusual in *LITAF*-related CMT1C. After the mutation was detected in an unaffected sister, further family analysis was performed with NGS, which revealed 2 compound heterozygous pathogenic mutations in *SH3TC2* (c.2860C>T p.[Arg954Ter] and c.3303delG p.[Arg1101SerfsTer15]), which segregated with the disease in the family and led to reclassification of the c.115C>T p.(Pro39Ser) in *LITAF* as a VUS.

Mutations in *MFN2*, including one case with 2 recessive variants, a c.449G>T p.(Gly150Val) missense variant in compound heterozygous state with a deletion of exons 7–8, and one previously reported case^26^ with 2 semidominant c.749G>A p.(Arg250Gln) and c.1085C>G p.(Thr362Arg) missense mutations, accounted for 12% of all CMT cases and 37% of axonal CMT cases who received genetic confirmation. Onset was variable, ranging from 3 to 52 years, and the severity ranged from mild to severe, with higher disability observed in a case carrying 2 semidominant mutations.

Six patients had mutations in *MPZ*, including 4 missense mutations, 1 mutation affecting a splicing site, and 1 whole-gene duplication. Three cases had onset in the first 2 years of life with delayed walking and slow or unrecordable nerve conduction velocities. Three additional cases had adult onset including 2 with axonal neuropathy of moderate severity.

Four patients had mutations in *GDAP1*. Two patients with recessive mutations in *GDAP1* presented with an early onset, severe neuropathy associated with vocal cord paralysis. Motor action potentials were not recordable. Two patients carrying single missense mutations had a milder axonal neuropathy with later onset. Pyramidal signs were present in one case.

Four patients were found to have homozygous or compound heterozygous variants in *IGHMBP2* causing severe autosomal recessive axonal CMT with early onset. Two unrelated cases carrying the same c.1325A>G p.(Tyr442Cys) homozygous variant and both from the Middle East had associated respiratory failure and recurrent gastric distention. In one patient, which has been previously reported, this was associated with hyperhidrosis of the hands and feet.^27^

Probable pathogenic mutations in *LRSAM1* were identified in 3 CMT2 cases from London. All mutations were inside or in close proximity to the RING finger domain, where all previous pathogenic dominant mutations have been reported. Of note, 2 had prominent vibratory sense loss in the lower limbs.

Three patients with mutations in *FDG4* presented with early-onset moderate to severe autosomal recessive CMT1 associated with scoliosis and cranial nerve involvement and very slow conduction velocities in the region of 10 m/s.

Five cases with dominant axonal CMT or dHMN had mutations in tRNA-synthetase genes: 3 in *GARS* and 2 in *AARS*. Two patients with *GARS* mutations presented with the characteristic hand weakness and atrophy.

## Discussion

This study provides evidence that targeted NGS panels are a useful tool for the molecular diagnosis of CMT in a clinical setting and are able to diagnose a third of patients not carrying the 17p duplication.

Previous studies have shown that NGS panels are technically robust in terms of coverage and read depth and have reported rates of molecular diagnosis in inherited neuropathies ranging from 6% to 46%.^10–19^ The differences in the diagnostic rate in these studies both between each other and compared to ours may be explained by the differences in the specific features of the cohorts being tested coming from general neurology, genetic or specialized inherited neuropathy clinics, the number of demyelinating CMT cases enrolled, and the variable exclusion of more common causative genes by previous MLPA and Sanger sequencing. As opposed to demyelinating cases, over 70% of axonal CMT cases remain genetically unconfirmed after NGS panel testing. NGS panels only explore a very limited part of the coding genomic DNA. Recent studies have shown that a significant part of the missing heritability in neurologic diseases as well as hereditary neuropathies may be hidden in noncoding regions of the human genome.^28–31^ The increased identification of mutations in noncoding DNA regions will likely lead to a reduction of the percentage of patients without a molecular diagnosis.

Of interest, 3% of patients who underwent NGS panel testing had copy number variants in one of the CMT-causing genes. Previous reports also demonstrated the ability of NGS to identify duplications and deletions in chromosome 17p12, as well as copy number variants in other genes.^14,17^ Besides the common *PMP22* rearrangements, pathogenic copy number variants are known in *MPZ*, *GJB1*, *MFN2* (in compound heterozygous state with a second pathogenic mutation), *NDRG1*, *GAN*, and *SEPT9*.^32,33^ More recently, a 78-kb duplication of chromosome 8q24.3 locus at chromosome Xq27.1 and a 1.35-Mb duplication of chromosome 7q36.3 were identified as the cause of *CMTX3* and *dHMN1*, respectively.^32^ Our study identified novel pathogenic copy number variants in *FDG4* and *SH3TC2* and suggest that implementation of NGS panels in a diagnostic setting will lead to an increased identification of structural variants in known CMT genes.

In contrast to most previous studies, we aimed at evaluating the accuracy of NGS panels in a real-life clinical specialist setting. We prospectively enrolled 220 patients accessing specialist clinics for CMT in the United Kingdom and United States and for whom NGS panels had been requested as a diagnostic test. We reviewed their genetic and clinical data upon completion of the diagnostic process in a multidisciplinary setting as this is a crucial step in modern genetic diagnostic practice.

This study confirms that mutations in *GJB1*, *MFN2*, and *MPZ* account for a significant proportion (39%) of genetically confirmed cases of CMT as we and others had shown in previous studies using sequential Sanger testing.^4,5^ The remaining 61% of genetically diagnosed cases encompass mutations in various less common genes. It is interesting to note that mutations in these genes accounted for only 6%–10% of genetically confirmed cases in our 2 previous studies, which looked at the prevalence of CMT genetic subtypes in the London and Iowa (previously Detroit) cohorts based on traditional Sanger sequencing.^4,5^ Although a direct comparison is not possible, as some of the genes here identified were not known at that time, this would correspond to an increase of the diagnostic yield for these rarer genetic subtypes by 6- to 10-fold, thus highlighting the effectiveness of unbiased NGS panel testing in the diagnosis of CMT. As per our inclusion criteria, those percentages do not completely reflect the distribution of subtypes and mutations in the CMT population, since PMP22 duplication and deletions were excluded prior to enrollment in typical demyelinating cases, leading to a reduced inclusion of these patients compared to those with axonal/intermediate CMT subtypes.

Of note, in Iowa, mutations in one of the 3 common genes (*GJB1*, *MFN2*, *MPZ*) explained over 65% of positive cases vs only 18% in London. The discrepancy is likely due to differences in the baseline features of patients enrolled. In London, a significant number of patients had previously been tested for mutations in these genes by Sanger sequencing as this is available freely throughout the United Kingdom in the National Health Service in neurology and genetic clinics and, if present, NGS testing was not requested. This may have led to a selection bias in the London cohort towards the enrollment of rarer genetic subtypes, while the frequency of mutations identified in Iowa may reflect more reliably their actual prevalence in the general CMT population. Differences in the ethnicities of patients enrolled in the 2 centers may have also skewed the mutational spectrum towards rarer subtypes in the London population.

In both cohorts, and in keeping with previous reports,^34–36^ *SH3TC2* appears to be a common cause of autosomal recessive CMT in the Caucasian population, explaining over one-fourth of all demyelinating cases after exclusion of the *PMP22* duplication/deletion, and followed by *FDG4*. Recessive mutations in *GDAP1* and *IGHMBP2* were also frequently identified in early-onset and severe axonal CMT cases. Three novel mutations and one novel VUS in *LRSAM1* were identified in the London cohort in patients with moderately severe axonal neuropathy and prominent sensory loss. Mutations in tRNA synthetase genes, *GARS* and *AARS*, were also well-represented among the identified genetic subtypes. A VUS in *MARS* was identified in one case whose neuropathy was otherwise explained by a likely pathogenic mutation in *LRSAM1*, while no cases with mutations in *HARS*, *YARS*, or *KARS* were identified.

A positive family history for neuropathy in dominant or X-linked cases and consanguineous marriages in recessive cases, an early age at onset of the neuropathy, and the presence of reduced conduction velocities were found to be independent predictors of positive testing by NGS panels. In fact, only 4/55 (7%) sporadic axonal or intermediate CMT cases with onset after 20 years received genetic confirmation in the present study compared to 17/18 (94%) early-onset familial demyelinating CMT cases tested. The percentage of genetically confirmed cases dropped to 5% in patients with atypical features or for whom a nongenetic cause of the neuropathy could not be ruled out. This is in keeping with previous studies showing that the age at onset is a strong predicting factor for obtaining a genetic diagnosis.^11^

The average price of an NGS panel covering 20 genes in the United Kingdom is £800 (∼$1,000 USD), corresponding to an average cost of £40 per gene. Considering that the traditional Sanger sequencing price ranges from £200 to £500, so over 10-fold higher, NGS panels appear to be effective both from a diagnostic as well as from an economic point of view.

While NGS sequencing is able to sequence multiple genes in a fraction of the time compared to sequential Sanger sequencing, the time and expertise required for the analysis of the many variants identified has greatly increased. In particular, the interpretation of VUS remains one of the largest diagnostic challenges in the NGS era. Misclassification of a VUS as causative or benign may have major clinical and legal implications, especially if one thinks of the available family planning options (e.g., prenatal or preimplantation diagnosis) that patients may undertake after receiving a molecular diagnosis. In our study, VUS were identified in approximately one-third of tested cases. Single variants in genes associated with CMT in a recessive state were by far the most common type of VUS. Although their interpretation as not causative by themselves of the neuropathy may seem relatively straightforward, they require careful consideration as large rearrangements or variants in promoter/noncoding regions on the other allele could be missed by both NGS and Sanger sequencing, as exemplified by 2 cases here reported carrying point mutations in *SH3TC2* or *FDG4* in one allele and a larger rearrangement on the other allele. Introducing whole genome analysis into clinical practice will greatly assist the analysis of these cases.

Of interest, a previous study had identified that the number of rare VUS in neuropathy-associated genes, including single mutations in autosomal recessive CMT genes, was significantly higher in patients with CMT vs unaffected controls.^9^ These variants were shown to interact genetically in a zebrafish model, exacerbating their phenotype. The authors concluded that the combinatorial effect of rare variants could contribute to the disease burden in CMT and partly explain its variable phenotypic expressivity. Taking advantage of the large dataset collected in this study, we analyzed our data to see whether the presence of additional VUS could aggravate the phenotype in genetically confirmed patients. We did not observe any significant differences in disease severity in solved cases carrying additional VUS vs those without a second variant. The genetic heterogeneity of the cohort examined and the relatively low number of patients per single genes could be a major limiting factor in the interpretation of the negative finding, as a VUS could act as a modifier for a particular CMT subtype but not others.

Finally, we have confirmed that NGS can accurately detect copy number changes at the whole gene as well as single exon level, both for *PMP22* as well as in other genes including *MPZ*, *MFN2*, *SH3TC2*, and *FDG4*. To our knowledge, copy number variations in *SH3TC2* and *FDG4* were not previously reported as causative of CMT. This finding highlights how structural variants in known CMT genes could potentially account for part of the current missing heritability in CMT.

Our study has shown that, after exclusion of the common *PMP22* duplication/deletion, NGS panels can achieve a molecular diagnosis in one third of CMT cases tested in a specialized clinical diagnostic setting, which includes a dedicated post genetic analysis multidisciplinary case discussion. Sixty percent of the genetically confirmed cases carried variants in heterogeneous and often very rare genes, whose identification would have been extremely laborious by direct sequencing of candidate genes. Early disease onset, a positive family history or consanguinity, and reduced nerve conduction velocities were predictive of achieving a positive genetic diagnosis.

## Glossary

CMT: Charcot-Marie-Tooth disease
CMTES: CMT examination score
CMTNS: Charcot-Marie-Tooth neuropathy score
dHMN: distal hereditary motor neuropathy
HSN: hereditary sensory neuropathy
MLPA: multiplex ligation-dependent probe amplification
NGS: next-generation sequencing
NHNN: National Hospital for Neurology and Neurosurgery
VUS: variants of unknown significance
WES: whole exome sequencing
